# Myelodysplastic Syndrome Clinically Presenting with the “Classic TTP Pentad”

**DOI:** 10.1155/2017/4619406

**Published:** 2017-02-01

**Authors:** Santiago Fabián Moscoso Martínez, Evelyn Carolina Polanco Jácome, Elizabeth Guevara, Vijay Mattoo

**Affiliations:** ^1^Department of Hematology and Oncology, The Brooklyn Hospital Center, 121 Dekalb Ave, New York, NY 11201, USA; ^2^Department of Pathology, Hofstra Northwell Health School of Medicine, 6 Ohio Drive, New Hyde Park, NY 11042, USA

## Abstract

The clinical presentation of myelodysplastic syndrome (MDS) is not specific. Many patients can be asymptomatic and can be detected only due to an abnormal complete blood cell count (CBC) on routine exam or for other reasons while others can be symptomatic as a consequence of underlying cytopenias. Thrombotic thrombocytopenic purpura (TTP) usually is suspected under the evidence of microangiopathic hemolytic anemia (MAHA) and thrombocytopenia and because it is a life-threatening condition (medical emergency) immediate initiation of plasmapheresis could be life-saving. The following case illustrates an unusual presentation of MDS in a patient who came in to the emergency room with the classic TTP “pentad” of fever, renal involvement, MAHA, mental status changes, and thrombocytopenia. We will focus our discussion in the clinical presentation of this case.

## 1. Introduction

MDS is a clonal stem-cell disorder characterized by dyshematopoiesis and dysplasia of single or multiple blood cell lines causing progressive cytopenias. As a result, MDS, if symptomatic, is manifested with signs and symptoms related to the severity of the underlying cytopenias (e.g., fever, infection, pallor, fatigue, petechiae, ecchymosis, bleeding, and hematomas). However, MDS in an elderly patient presenting as full-blown TTP is unusual. We present a case of a sixty-six-year-old woman coming to the emergency room with the classic TTP “pentad” and ended up with a diagnosis of MDS.

## 2. Case Report

A 66-year-old African American female without significant personal or family medical history and who has not been followed by any physician for years came in to the emergency department due to acute onset altered mental status changes. As per patient's daughter, she was in good health until the night prior to admission after a two-mile walk. In the morning she was noticed to be drowsy and confused “trying to drink her hair as if it were a glass of water”. She could not identify her daughter and she was complaining of generalized weakness as well as right groin pain. As the morning went by she was progressively getting worse becoming somnolent and stopped responding to verbal commands. She was brought to the emergency room where she was found to be febrile (as high as 106 degrees Fahrenheit), tachycardic (145 beats per minute), and tachypneic (25 breaths per minute) with a normal blood pressure. She was only responsive to painful stimuli with no meningeal signs. No palpable organomegaly or cervical, supraclavicular, axillary, or inguinal lymphadenopathy was found.

**Figure 1:**
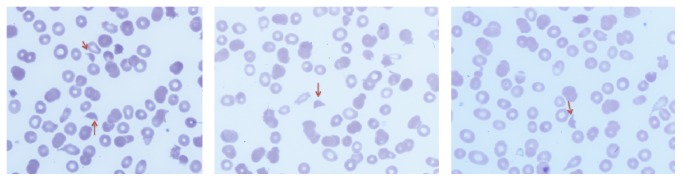
Peripheral blood smear shows fragmented helmet shaped rbcs (red arrows) (2-3 per high power field).

**Figure 2:**
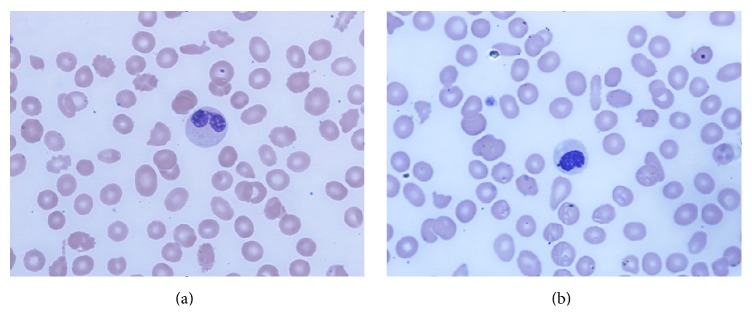
Peripheral blood smear (Wright Giemsa stain at 100x magnification) shows dysplastic neutrophils characterized by nuclear hypolobation (Pseudo-Pelger Hüet) and decreased granules (a). Neutrophil with a nonlobated nucleus (b).

Complete blood cell count showed normocytic anemia (Hb 6.6 g/dL; MCV 91), thrombocytopenia (platelet count 77,000), and slight leukocytosis with neutrophilia (WBC 13,400. ANC 11,800). Further testing revealed an elevated LDH 1248 U/L (125–220 U/L), reticulocyte count 3.5% (1-2%), mild indirect hyperbilirubinemia (total bilirubin 1.4 mg/dL and direct bilirubin 0.3 mg/dL), and mildly elevated creatinine (1.2 mg/dL). Vitamin B12 level, folate level, prothrombin time, partial thromboplastin time, and INR were all normal as well as fibrinogen level (402 mg/dL). Serum iron was 6 mcg/dL (50–170 mcg/dL), total iron binding capacity was 257 mcg/dL (179–378 mcg/dL), and ferritin was 378 ng/mL (10–204 ng/mL). Urinalysis showed microscopic hematuria and proteinuria (30 mg/dL). Antibodies to human immunodeficiency virus were negative. Peripheral smear showed marked schistocytosis (Figure 1) and dysplastic neutrophils (Figures 2(a) and 2(b)).

Chest X-ray and computed tomography (CT) of the head were unremarkable. Because of the compelling evidence of microangiopathic hemolytic anemia (MAHA) associated with thrombocytopenia, alter mental status, fever, and renal involvement thrombotic thrombocytopenic purpura (TTP) was strongly considered. ADAMTS-13 was sent and she was started on daily plasmapheresis and steroids. Platelet count dropped even further in the next couple of days (nadir 9,000) and there was no improvement in the MAHA (including the persistent marked schistocytosis) Table 1. ESR was 5 (normal range: 0–20 MM/HR). Three days after the beginning of plasmapheresis the test results for haptoglobin as well as for ADAMTS-13 activity came back and they were 120 (14–250 mg/dL) and 52% (normal: 68–163%), respectively. Plasmapheresis was stopped. Urine culture and blood cultures were negative for infection. CT chest, abdomen, and pelvis with contrast as well as CT angiogram of the lower extremities were unremarkable. Flow cytometry showed no evidence of B-cell lymphoma, T-cell lymphoma, acute leukemia, or increase in blasts.

**Figure 3:**
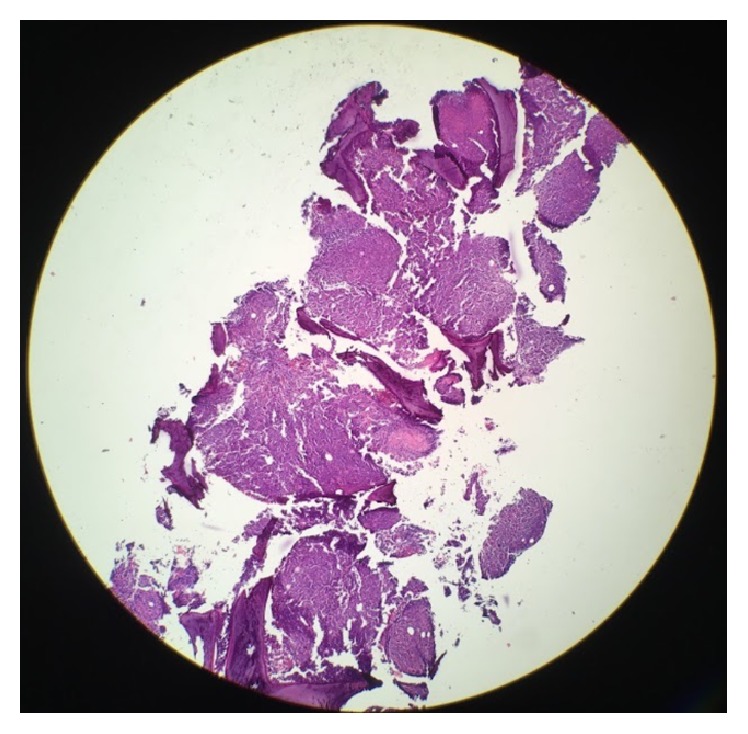
Bone Marrow Core Biopsy: low power magnification, highly cellular bone marrow.

**Figure 4:**
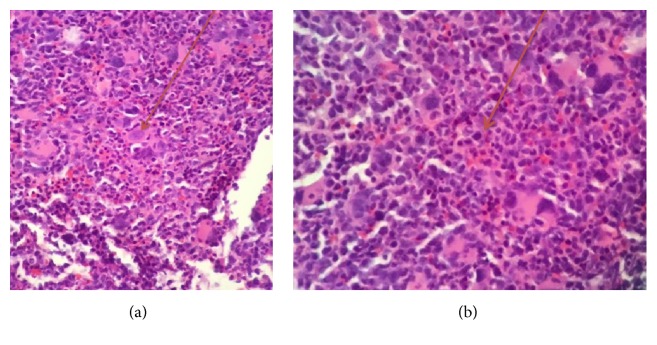
Bone Marrow Core Biopsy: picture (intermediate magnification, H&E) shows a hypercellular bone marrow with increased number of dysplastic megakaryocytes. Dysplastic features include nuclear hypolobation (a) and micromegakaryocytes (b).

**Figure 5:**
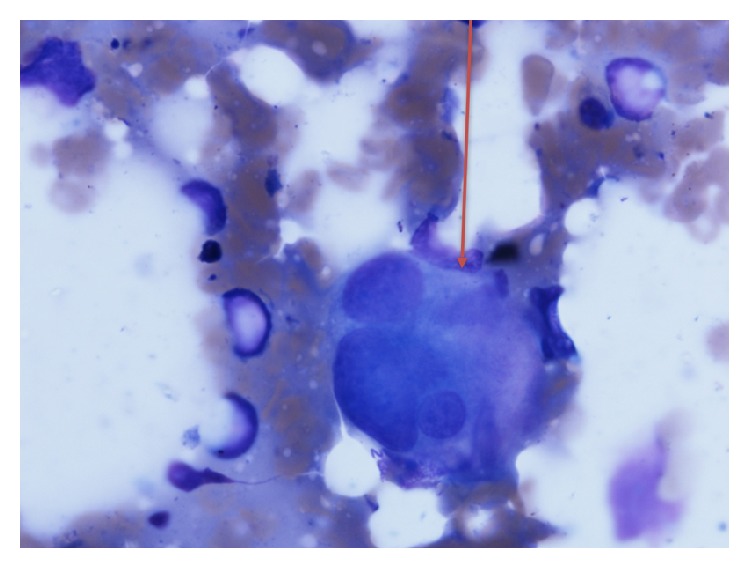
Bone marrow aspirate smear: dysplastic megakaryocytes, 100x magnification multinucleated megakaryocyte.

**Figure 6:**
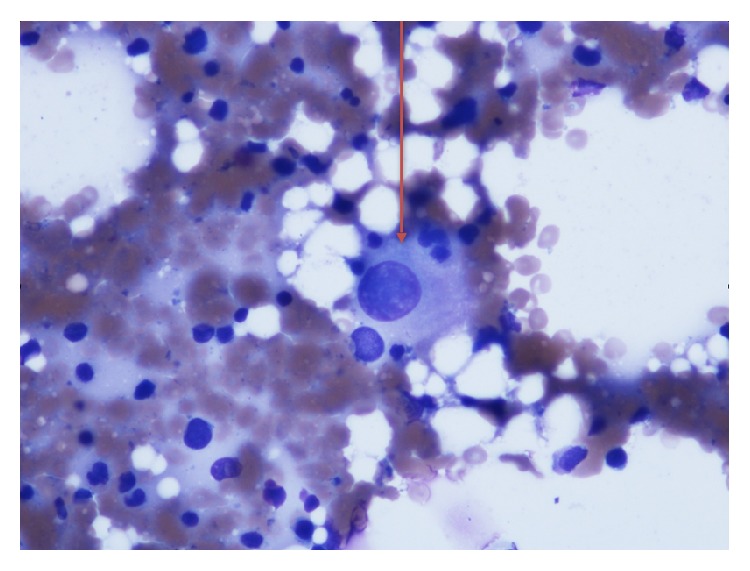
Bone marrow aspirate smear: dysplastic megakaryocyte, 100x magnification megakaryocyte with nuclear hypolobation.

**Figure 7:**
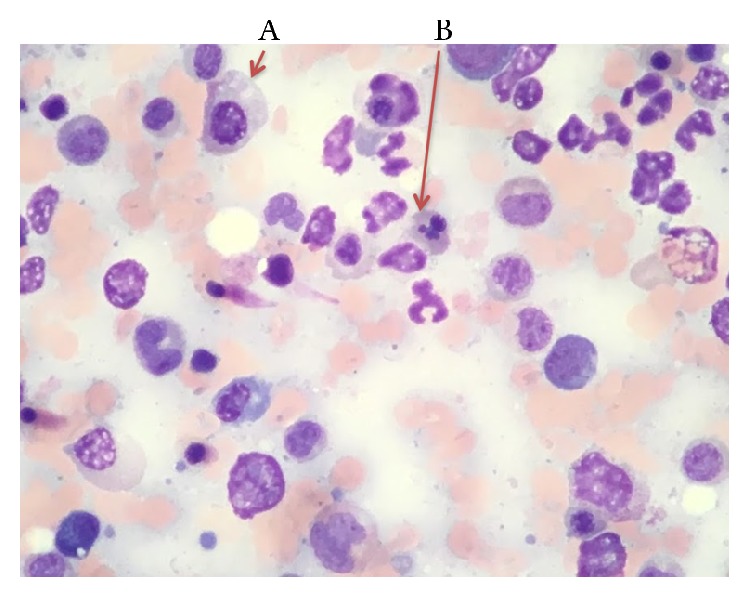
Bone marrow aspirate shows dyserythropoiesis including megaloblastic change (A) and nuclear budding (B).

She underwent to bone marrow aspiration and biopsy which showed markedly hypercellular marrow, hyperplastic and dysplastic megakaryocytic, and dyspoietic erythroid elements with occasional binucleate forms and irregular contour, and myeloid elements showed slight left shift. There was mild increase in reticulin fiber content making a myelophthisic process as the underlying cause of schistocytosis unlikely (Figures 3–7). There was no evidence of plasma cell disorder. Iron stain on biopsy showed mild increase in stainable iron (3+) and no ring sideroblasts. There was no increase in blasts.

**Figure 8:**
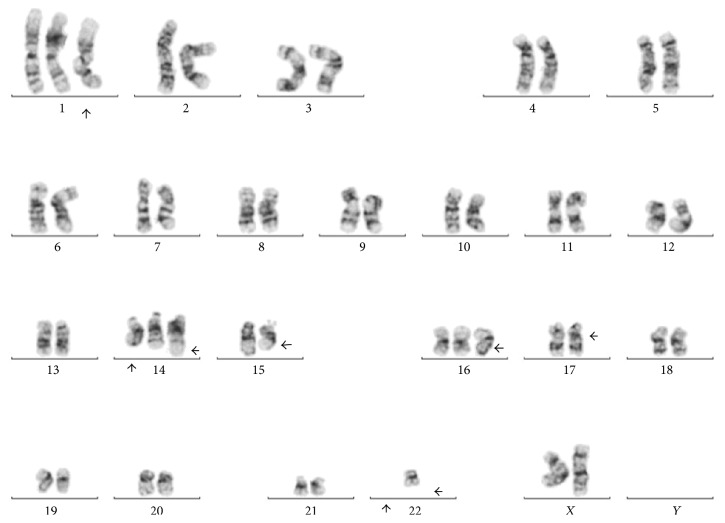
Bone marrow specimen showing 48,XX,+1,+14,t(14;22)(q32;q11.2),add(15)(q22),+16,add(16)(q12-13),add(17)(p11.2),−22,inc.

**Figure 9:**
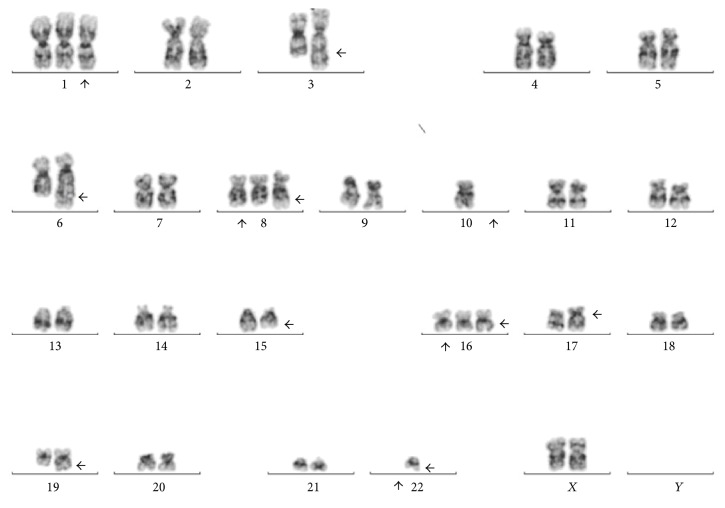
Bone marrow specimen showing 47,XX,+1,add(3)(q21),der(6)t(6;10)(q25;q11.2),+8,t(8;22)(q24.1;q11.2),−10,add(15)(q22),+16,add (16)(q12-13),add(17)(p11.2),add(19)(q13.1),−22.

Karyotype analysis in twenty metaphases was performed and revealed a complex karyotype in eight metaphases (Figures 8 and 9). Twelve metaphases were chromosomally normal. Due to all these findings the final diagnosis was MDS with multilineage dysplasia [1] and she was referred for a clinical trial.

## 3. Discussion

This is a case of an elderly patient with MDS clinically presenting with the “classic TTP pentad.” To the best of the authors' knowledge this is the first case reported of MDS presenting as full-blown TTP in an elderly person. There are three cases in the twenties reporting the association between TTP and MDS [2–4]. Our case illustrates that MDS can clinically present as TTP and life-saving treatment such that plasmapheresis in this acute clinical setting should not be delayed until further testing to clarify the diagnosis is in process.

MDS generally affects older adults (median age 76 years) [5]. Clinically MDS can be incidentally found in a work-up done for other reasons (CBC showing anemia, leukopenia, or thrombocytopenia) and being asymptomatic usually in half of the cases [6] or it can be symptomatic with clinical features related to the severity of the underlying cytopenias which in turn are due to ineffective hematopoiesis.

At least 80–90% will have anemia at the time of diagnosis; it is usually normocytic or macrocytic, and half of them will have an Hb less than 10 g/dL [7]. Anemia can be manifested as fatigue, generalized weakness, exercise intolerance, angina, dizziness, or alter mental status [8–11]. In the present case her Hb on admission was 6.6 g/dL explaining her fatigue, generalized weakness, and at least in part her alter mental status.

Thrombocytopenia is present in 30–45% of cases and approximately 40% are neutropenic at the time of diagnosis [7]. As a result of them and less often patients can have petechial rash, easy bruising, inappropriate bleeding, hematomas, or infection. Our patient did not have clear evidence of infection by physical exam, cultures, and imaging studies. After approximately a week she was found to have a right thigh abscess that was drained. The cause of fever appears to be due to underlying infection. Weight loss is rare and represents a late manifestation of MDS.

On physical exam there are not specific findings related to MDS. Pallor is seen in 60% of the cases and rash and purpura in 26%. Lymphadenopathy, splenomegaly, and hepatomegaly are uncommon [9, 12].

MDS can be suspected when there is unexplained monocytosis, cytopenias, macrocytosis (even with normal hemoglobin), or cellular atypia and dysplasia. In our case the presence of cytopenias and cellular dysplasia were suggestive of MDS.

Peripheral blood smear findings described in the erythroid line in MDS include spherocytes, macroovalocytes, acanthocytes, elliptocytes, stomatocytes, teardrop cells, nucleated erythrocytes, basophilic stippling, and howell-jolly bodies. However, schistocytes have been rarely described and to the best of our knowledge, only 4 cases have been reported in the English language literature [13–15]. In the present case, schistocytosis could be due to an underlying disseminated intravascular coagulation (DIC) perhaps due to an underlying infection (thigh abscess).

The initial presentation of acute onset and rapidly progressive worsening alter mental status changes, fever, anemia with marked schistocytosis (MAHA), thrombocytopenia, and renal involvement made TTP occupy the top in the list of the differential diagnosis in this unusual case.

Diagnosis of MDS relies on the morphological assessment of bone marrow aspiration and biopsy as well as on the cytogenetic analysis. In some occasions bone marrow aspiration and biopsy need to be repeated more than once since MDS can affect the bone marrow unevenly and it can cause false negatives.

## 4. Conclusion

In an elderly patient who presents with anemia and marked schistocytosis (microangiopathic hemolytic anemia) associated with thrombocytopenia MDS should be considered in the differential diagnosis. As illustrated in the present case, MDS can even present with the classic TTP “pentad” of fever, renal involvement, anemia (MAHA), mental status changes, and low platelets (thrombocytopenia). In this clinical context, MDS becomes a diagnosis of exclusion and life-threatening conditions such that TTP needs to be addressed first with appropriate evaluation and initiation of pertinent treatment until this medical emergency is being ruled out.

## Figures and Tables

**Table 1:** Schematic representation of hemoglobin, platelets, schistocytes, and plasmapheresis.

	Day 1	Day 2	Day 3	Day 4
Hemoglobin (g/dL)	6.6	6.8	7.7 (transfused one unit)	7.0
Platelets (K/cmm)	77	50	12	9
Schistocytes	Marked	Marked	Marked	Marked
Plasmapheresis	Started	Continued	Continued	Stopped
